# The Book World of Medicine and Science

**Published:** 1904-12-17

**Authors:** 


### The Book World of Medicine and Science.

Construction of Hospitals for Consumption. By John W. Hayward, M.D. (Liverpool: Egerton Smith and Co. Pp. 26. Price 1s.)

As yet the construction of sanatoria for consumptives is not much more than in its infancy. At all events, it has not reached adolescence—much less maturity; and therefore any contribution coming from an expert is deserving of attention. We cannot say that we approve of everything which Dr. Hayward has to tell us, but some of his observations are well worthy of acceptance. We are entirely at one with him when he says consumption hospitals should be as carefully built as other hospitals for infectious diseases, but when he says that "all the floors, landings, stairs, and such-like portions should be of granolithic," and that "all the walls should be lined with glazed bricks, or tiles, or enamelled slate slabs," we think he is running into somewhat unnecessary expense. It is more than probable that the sanatorium of the future will consist of an administrative block or centre, a hospital ward or two for acute cases, and a series of quite inexpensive wooden châlets adapted for one, two, or at most four beds. These cubicles would have perfect cross-ventilation by windows, doors, or fanlights on all the four sides, so that pure air would blow freely through them. In Dr. Hayward's plan, the rooms, all single-bedded, are arranged on both sides of a corridor. In our opinion this is a faulty arrangement. Cross-ventilation of the rooms is most difficult, and we fail to see any compensatory advantage in the system. Dr. Hayward boldly says that cross-ventilation is unnecessary in our climate, and with this dictum we absolutely disagree. Again, as regards warming of hospitals, he does not believe much in open fireplaces, and tells us "they are of but little use as means of ventilation." Nevertheless, there is a great deal to be said in favour of the open fireplace. We may breathe air of almost any degree of coldness with impunity provided the skin be kept warm by plenty of clothing. The fact is that the rays of heat from an open fire resemble those from the sun inasmuch as they pass through the air without warming it, while hot water or other radiators actually warm the air, and to warm the body with the air we breathe cannot be good for a healthy man, and is worse still for a sick man. It is perhaps true that in our fickle climate radiators may be occasionally used without much danger to the sick, but their use must be jealously guarded, and never solely relied on: they must never be more than an adjunct. We have pointed out some objectionable points in Dr. Hayward's pamphlet, but it must not be supposed that we should adversely criticise the whole of it. On the contrary, there are many useful hints, and we regret that want of space prevents our noting them.

Guide to the Examination of the Throat, Nose, and Ear. By William Lamb, M.D. Edin., M.R.C.P. Lond., Honorary Surgeon, Birmingham Ear and Throat Hospital. (London: Baillière, Tindall & Cox. 1904. Pp. 152. Price 5s. net.)

This book is intended for senior students and practitioners. It is essentially practical. Dr. Lamb writes clearly and to the point. He records what he knows from long personal experience. He anticipates difficulties—nearly all the difficulties likely to be met with in examining these special organs. Separate chapters are devoted to the management of the light and reflector; to the examination of the mouth and pharynx, including the naso-pharynx; of the nose, and of the larynx; to the commoner morbid alterations in the laryngoscopic image; to the examination of the ear; and to hints on local treatment. The author in his preface says he has aimed at being extremely elementary. After carefully sketching the normal topography and appearances of each region, he gives a brief outline of the principal abnormal conditions likely to be met with. Although elementary, it is so only in a comparative sense, for it is thorough, and in its thoroughness it differs from the ordinary elementary text-books of medicine. Practitioners skilled in the surgery of these special organs will not fail to derive considerable satisfaction, and also, we venture to think, much useful information, from a perusal of this little book. The chapter on nasal diseases is especially worth studying. The writer simplifies a large section of nasal pathology by regarding the ethmoid as the key of the nose. The various forms of ethmoiditis, with and without suppuration, constitute by far the most important of nasal diseases. The student will find the book most helpful in note-taking in the special departments. We can, therefore, well recommend this book to those interested, as well as to those wishing to become interested, in the modern surgery of the throat, nose, or ear.

*X*-Rays: Their Employment in Cancer and other Diseases. By R. J. Cowen, L.R.C.S.I., L.R.C.P.I. (London: 1904. H. J. Glaisher. Pp. 129. Price 2s. 6d. net.)

The beneficial effects of *x*-rays in the treatment of various morbid conditions, including some forms of malignant disease, is now beyond question, and, as the author remarks, it is equally unwise at the present time to dogmatically limit their application or to regard them as a panacea for all human ills. Advance in knowledge of their use must be sought in the collection and correlation of reports combining details of the therapeutic measures employed with accurate information as to the conditions of the disease under treatment. In the cases recorded by the author the former of these conditions is well observed, but he shows distinctly less appreciation of the other factor in the problem. This is specially to be regretted in the chapters on malignant disease where information of this kind is quite as necessary as is that bearing on the electrical conditions. The clinical descriptions of the tumours are inadequate, both as to their symptoms and connections, while their pathological nature is often insufficiently indicated. The author's views as to the use of *x*-rays as a substitute for, or supplement to, surgical operation for cancer are scarcely likely to be generally adopted, but his experience of operation seems to have been unfortunate and his estimate of its value correspondingly low. In a second edition such expressions as tumours of the "os" uteri, "the size of a large potato," or "speculi" might well be amended.

